# Anatomical and functional results of plasma rich in growth factors as a treatment for poor-prognosis macular holes

**DOI:** 10.22336/rjo.2025.81

**Published:** 2025

**Authors:** Marta Caminal-Caramés, Jaume Crespí Vilimelis, Carlos Oribio Quinto, Albert Saladrigas Pernias, Santiago Conversa, Alejandro Verdú Reyes, Jesús Díaz Cascajosa, Daniela Rego Lorca, Maria Pilar Piquer Pérez, José Ignacio Vela Segarra

**Affiliations:** 1 Department of Ophthalmology, Hospital de la Santa Creu i Sant Pau, Barcelona, Spain

**Keywords:** full-thickness macular holes, PRGF, ILM peeling, inverted ILM flap, AS = autologous serum, BCVA = best corrected visual acuity, ELM = external limiting membrane, EZ = ellipsoid zone, FTMH = full-thickness macular hole, GF = growth factors, ILM = internal limiting membrane, iPRGF = injectable plasma rich in growth factors, OCT = optical coherence tomography, PPV = pars plana vitrectomy, PRGF = plasma rich in growth factors, PRP = platelet-rich plasma, RPE = retinal pigment epithelium, SD-OCT = spectral-domain optical coherence tomography

## Abstract

**Purpose:**

The management of poor-prognosis full-thickness macular holes (FTMH), considered as large, myopic, recurrent, or persistent, is challenging, and there is no consensus on the most suitable option. Plasma rich in growth factors (PRGF) is a subtype of platelet-rich plasma with a higher concentration of growth factors, which promotes Müller cell proliferation and, therefore, closure of FTMH. This study aims to analyze the benefits of injectable plasma rich in growth factors (iPRGF) as an adjuvant to PPV in poor-prognosis FTMH.

**Materials and methods:**

A prospective, non-randomized study was conducted in patients with poor-prognosis FTMH. Patients were treated with PPV associated with adjuvant iPRGF. Postoperative best corrected visual acuity (BCVA), FTMH closure, retinal layer restoration, and type of closure were analyzed.

**Results:**

A total of 12 eyes were included in the study. Complete closure of the FTMH was achieved in 92% of the patients, and BCVA improvement in 75%. Reconstitution of the external retinal layers was observed in 41% of the patients, and closure type 1A in 41%. No adverse effects were recorded.

**Discussion:**

The high closure rate and visual improvement suggest that iPRGF is a promising adjuvant in poor-prognosis FTMH, likely through Müller cell stimulation and glial-mediated tissue repair. Compared with more complex techniques such as inverted ILM flaps or autologous retinal transplants, iPRGF offers a more straightforward, safer, and faster approach, particularly in myopic or recurrent cases. The potential synergistic effect of combining iPRGF with ILM flap techniques warrants further investigation.

**Conclusion:**

The use of iPRGF may be an effective technique for achieving anatomical closure, restoring retinal layers, and improving final BCVA.

## Introduction

Full-thickness macular holes (FTMH) are a vitreoretinal interface pathology affecting the center of the macula, with a prevalence of 0.3-5% [**1**]. Most cases of FTMH are primary or idiopathic, due to vitreomacular traction, and occur more frequently in women after the sixth decade of life [**2**]. Although the Gass classification system has historically been used for categorization, the current predominant classification is the International Vitreomacular Traction Study Group (IVTS), which categorizes FTMH based on minimum diameter as assessed by Optical coherence tomography (OCT) and by etiology or vitreous condition [**3**].

The most effective strategy for managing FTMH involves performing a pars plana vitrectomy (PPV), peeling the internal limiting membrane (ILM), and using tamponade gas. This method is highly successful, achieving closure rates of 85-100% [**4,5**].

However, there are subtypes of FTMH with poor prognosis, characterized by lower closure rates, such as large, myopic, traumatic, persistent, and recurrent FTMH. The reported closure rate for persistent or recurrent FTMH using the gold-standard surgical technique ranges from 46% to 69%. In other case series that included very large FTMH, high myopia, or a history of retinal detachment, the closure rate was even lower, ranging from 8 to 44% [**6,7**]. The management of these poor-prognosis FTMH is challenging, and although new adjuvant techniques have been proposed, there is no consensus on the optimal approach [**8**]. These techniques include ILM peeling enlargement, inverted ILM flap technique, ILM free flap, lens capsular flap, autologous neurosensory retinal transplantation (ART), human amniotic membrane (hAM), and blood-derived products, such as autologous serum (AS), autologous platelet-rich plasma (PRP), and plasma rich in growth factors (PRGF) [**9-13**].

PRGF is a subtype of PRP with a higher concentration of growth factors (GF). The rationale for its application in FTMH is that the high concentration of platelets and GF promotes tissue regeneration, modulates inflammation, and stimulates Müller cell proliferation, which could ultimately contribute to FTMH closure [**14**]. There are studies demonstrating successful outcomes with blood derivatives and PRP [**12,15,16**]. Nevertheless, there are only a few studies on the use of PRGF, and, therefore, a lack of evidence demonstrating its potential benefits [**14,17,18**]. Most existing studies on the topic have small sample sizes and use different forms of PRGF (membrane, injectable, or both). To date, there is no consensus on the protocol for this procedure [**14,17,18**]. This study aims to report the anatomical and functional outcomes of injectable PRGF (iPRGF) as an adjuvant to PPV for a cohort of patients diagnosed with poor-prognosis FTMH.

## Materials and methods

We performed a prospective non-randomized single-center study with all consecutive patients diagnosed with poor prognosis FTMH and treated with PPV and adjuvant iPRGF therapy. The study was conducted in accordance with the Helsinki Declaration and was approved by the Institutional Ethics Committee. All patient data were anonymized for analysis.

We included consecutive patients diagnosed with poor-prognosis FTMH, defined as idiopathic large FTMH (>400 microns of minimum diameter), myopic FTMH (considering those patients diagnosed with pathological myopia), traumatic FTMH, persistent FTMH (no closure after first PPV), and recurrent FTMH (reopening after first PPV). Cases of large or myopic FTMH that were concurrently recurrent or persistent were classified as the latter. Patients with pathological myopia were defined by the presence of typical myopic degenerative changes, especially in the posterior pole, according to the International Myopia Institute [**19,20**]. Patients who did not sign the informed consent form, had active infection, had low platelet counts (<60000), or were immunocompromised were excluded. Patients with retinal detachment, diabetic retinopathy, epiretinal membrane, or previous ocular surgery (except cataract or FTMH surgery) in the eye included in the study were also excluded.

We reviewed age, sex, medical and ophthalmologic history, FTMH etiology, best corrected visual acuity (BCVA) by Snellen and logMAR, slit lamp examination of the anterior segment, and dilated fundus examination. Spectral-domain OCT (SD-OCT) using Heidelberg Spectralis was performed. According to the IVTS classification [**3**] and using the caliper function, we measured the minimum diameter (the narrowest distance between edges), the base diameter (edge-to-edge distance at the level of the retinal pigment epithelium (RPE)), and the height (distance between the RPE and the ILM). The diameter hole index (DHI), tractional hole index (THI), and macular hole index (MHI) were also calculated. DHI is the ratio of the minimum diameter to the base diameter, THI is the ratio of height to minimum diameter, and MHI is the ratio of height to base diameter.

The time from diagnosis to surgery was recorded. After the surgical procedure, BCVA and SD-OCT were performed at 3 and 6 weeks, and then at 9 months. The anatomical status of the macula was evaluated, including the closure of the FTMH and restoration of the outer layers. The closing pattern of the FTMH was analyzed based on the classification proposed by Rossi et al. [**21**], in which type 0 corresponds to open FTMH, type 1A closed FTMH with reconstitution of all retinal layers, type 1 B closed FTMH closure with interruption of external retinal layers, type 1C closed FTMH with interruption of internal retinal layers. Type 2 FTMH closed with autologous or heterologous tissue that disrupts the natural layered anatomy.

**Fig. 1:**
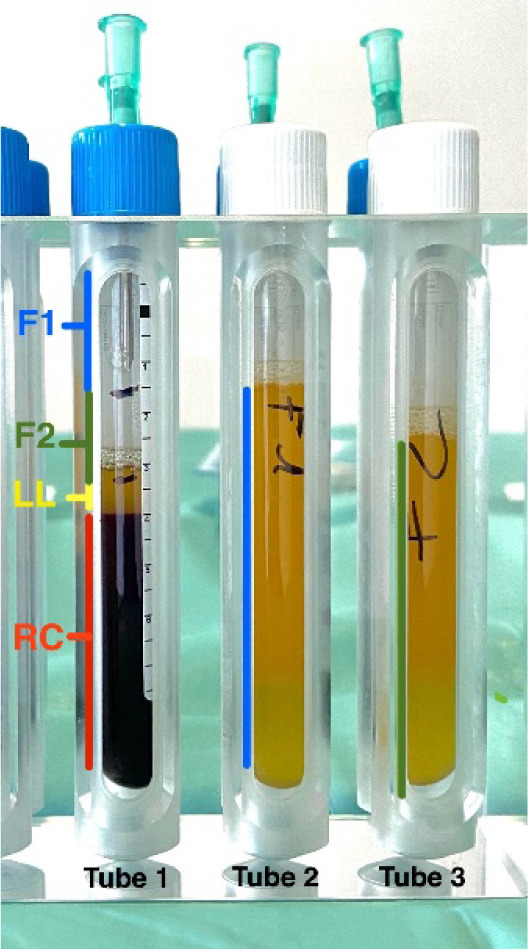
Preparation of plasma rich in growth factors by a 9mL tube of centrifuged peripheral venous blood. In the first tube, different fractions can be observed after centrifugation: Fraction 1 (F1), fraction 2 (F2), leukocyte layer (LL), and red cells (RC). F2 is the plasma with the highest platelet concentration, and F1 is the remaining plasma above. F1 and F2 have already been removed and placed separately in tubes 2 and 3, each containing 9 ml

PRGF was obtained following the protocol described by Anitua and coworkers [**22**]. Immediately before the start of surgery, under sterile conditions, 81mL of peripheral venous blood was collected in 9 mL tubes and mixed with 3,8% sodium citrate. By using Endoret closed system (PRGF, Ophthalmology kit, BTI Biotechnology Institute, S.L., Miñano, Álava, Spain), the blood was centrifuged at 580 g at room temperature for 8 min. This allowed the blood to be divided into different layers (from top to bottom): fraction 1 (F1), fraction 2 (F2), leukocyte layer, and red cells (**Fig. 1**). F2 is the plasma with the highest platelet concentration, and F1 is the remaining plasma [**12**]. 5mL of the F2 was collected with a sterile syringe and activated with 10% calcium chloride Endoret Activator (BTI Biotechnology Institute, S.L., Miñano, Álava, Spain) to prepare iPRGF.

All patients underwent three-port PPV surgery conducted by three experienced vitreoretinal surgeons (J. C., J. V., and J. D.). Most procedures were performed using 27- or 25-gauge vitrectomy with the Eva System (DORC, Zuidland, The Netherlands). When ILM was present, a standard peeling technique was employed with the assistance of VisionBlue® (DORC, Zuidland, The Netherlands). In patients with large idiopathic FTMH, an inverted ILM flap was combined with iPRGF to promote stabilization and regeneration.

After fluid-air exchange, three drops of iPRGF were gently added as an adjuvant over the fovea using an intraocular cannula, and the mixture was left to solidify for a few seconds. In all cases, 20% sulfur hexafluoride gas (SF6) was left as a tamponade. Throughout the postoperative period, patients were instructed to maintain a supine position for 30 min to ensure the PDGF clot remained in contact with the FTMH, followed by a prone position for 3 days.

Statistical analysis was performed using SPSS Software (version 22.0; SPSS Inc., Chicago, IL, USA). A descriptive study was performed, and measures of central tendency (mean and median) and dispersion (standard deviation and interquartile range) were determined based on the normal distribution of the variables. For quantitative variables, nonparametric tests (e.g., the Wilcoxon test) were used to evaluate changes before and after surgery. A scatter plot was used to compare pre- and postoperative BCVA. The results were considered statistically significant if the p-value was < 0.05.

## Results

Twelve eyes were included in the study. **Table 1** shows the demographic and clinical characteristics of patients before surgery. The basal characteristics of FTMH are summarized in **Table 2**. The sample consisted of 10 females (83%) and two males (16%). The mean age was 64.83 ± 10.4 years (Range: 48-81). Five patients were phakic before surgery (41%), and four patients had pathologic myopia (33%). Among the phakic patients, two underwent combined phacoemulsification and vitrectomy.

**Table 1:** Demographic characteristics, ophthalmological comorbidities, and macular hole etiology of patients

Patient	Age	Sex	Eye	Lens status	OA	FTMH etiology	Time to surgery (days)
1	81	Female	Left	Aphakic	Pathological myopia, glaucoma, trabeculectomy	Myopic	45
2	56	Female	Right	Pseudophakic	Pathological myopia, left RD	Recurrent and myopic	30
3	48	Female	Right	Pseudophakic	Pathological myopia	Myopic	30
4	58	Female	Left	Phakic		Persistent	15
5	66	Female	Right	Phakic		Large Idiopathic	30
6	57	Female	Right	Pseudophakic	Pathological myopia	Myopic	32
7	78	Male	Left	Pseudophakic	Retinitis pigmentosa	Persistent	30
8	76	Female	Left	Pseudophakic		Persistent	35
9	75	Male	Right	Pseudophakic		Large Idiopathic	40
10	62	Female	Right	Phakic		Persistent	30
11	58	Female	Left	Phakic		Large idiopathic	8
12	63	Female	Left	Phakic		Large idiopathic	35

RD = retinal detachment; OA = ophthalmic antecedents; FTMH = full-thickness macular hole; EM = epiretinal membrane

**Table 2:** Presurgical optical coherence tomography measures of macular holes included in the study

Patient	Base diameter (μm)	Minimum diameter (μm)	Height (μm)	DHI	THI	MHI
1	550	300	340	0.55	1.13	0.62
2	220	220	350	1.00	1.59	1.59
3	1700	370	690	0.22	1.86	0.41
4	1120	530	530	0.47	1.00	0.47
5	1270	870	410	0.69	0.47	0.32
6	780	310	360	0.40	1.16	0.46
7	300	300	170	1.00	0.57	0.57
8	1060	620	500	0.58	0.81	0.47
9	700	410	700	0.59	1.71	1.00
10	900	750	380	0.83	0.51	0.42
11	490	490	360	1.00	0.73	0.73
12	880	450	490	0.51	1.09	0.56
Mean (SD)	826.36 (442.5)	470 (206.01)	435.4 (158)	0.66 (0.26)	1.04 (0.49)	0.64 (0.36)
Median (IQ)	780 (630)	410 (320)	380 (180)	0.58 (0.53)	1 (1.02)	0.47 (0.31)
Range	220-1700	220-870	170-700	0.22-1	0.47-1.86	0.32-1.59

DHI = diameter hole index; THI = tractional hole index; MHI = macular hole index; SD = standard deviation; IQ = interquartile range

The poor-prognosis FTMH presented diverse etiologies: three were myopic (25%), one was recurrent (8%), four were persistent (33%), and four were large idiopathic (33%). Overall, seven patients (58%) had a large FTMH (>400 μm). The mean time to surgery was 29.9 +/- 15 days (Range: 8-45).

**Fig. 2:**
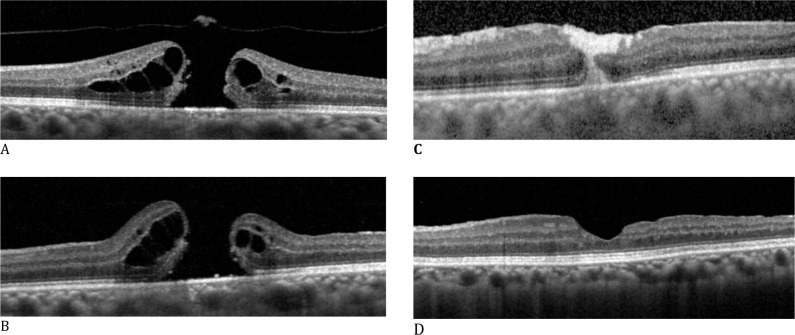
Persistent full-thickness macular hole (FTMH) with postoperative appearance of plasma rich in growth factors after 1 week of surgery and with complete anatomical closure after 9 months **A**. Preoperative optical coherence tomography (OCT) at diagnosis of FTMH; **B**. Postoperative OCT after the first surgery with pars plana vitrectomy (PPV) and internal limiting membrane peeling, in which closure was not achieved; **C**. Postoperative OCT after 1 week of PPV with adjuvant plasma rich in growth factors (PRGF). The clotted PRGF is noted as a hyperreflective material between the edges of the FTMH; **D**. Postoperative OCT after 9 months of surgery with type 1A FTMH closure

The anatomical and functional outcomes are presented in **Table 3**. The overall follow-up duration was 9 months. FTMH closure was achieved in 11 of the 12 eyes (92%). All patients with high myopia achieved FTMH closure. The only case in which closure was not completed was in patient 3 (recurrent FTMH), who had concomitant high myopia and a very large hole (1700 μm). The mean BCVA improved significantly from 0.1825 ± 0.12 (Range: 0.01-0.4) to 0.2967 ± 0.19 (Range: 0.001-0.6) at 9 months post-surgery (p=0.03; Wilcoxon test), as shown in **Table 4**. At the end of the follow-up period, BCVA improved in 9 patients (75%), worsened in 2 patients (16%), and remained stable in 1 patient (8%). Restoration of the outer layers was evaluated: type 1A was observed in 5 patients (41%), type 1B in 4 patients (33%), type 1C in 2 patients (16%), and type 0 in 1 patient (8%). **Fig. 2** shows FTMH type 1A of closure. No other complications were observed in our study.

**Table 3:** Best corrected visual acuity measures and macular hole postoperative evolution

Patient	PPV caliber	BCVA pre-surgical decimal (logMAR)	BCVA pre-surgical Snellen (m)	BCVA 6 weeks decimal (logMAR)	BCVA 6 weeks Snellen (m)	BCVA 9 months decimal (logMAR)	BCVA 9 months Snellen (m)	FTMH final closure	Restoration of outer layers	Type of closure
1	27-G	0.4 (0.39)	6/15	0.4 (0.39)	6/15	0.5 (0.3)	6/12	Yes	Yes	1A
2	27-G	0.36 (0.44)	6/19	0.3 (0.52)	6/19	0.5 (0.3)	6/12	Yes	Yes	1A
3	23-G	0.01 (2)	6/450	0.2 (0.69)	6/30	0.1 (1)	6/60	No	No	0
4	27-G	0.16 (0.79)	6/38	0.2 (0.69)	6/30	0.5 (0.3)	6/12	Yes	Yes	1A
5	27-G	0.05 (1.3)	6/120	0.2 (0.69)	6/30	0.2 (0.69)	6/30	Yes	No	1C
6	25-G	0.2 (0.69)	6/30	0.16 (0.79)	6/38	0.16 (0.79)	6/38	Yes	No	1B
7	25-G	0.2 (0.69)	6/30	0.001 (3)	<6/450	0.001 (3)	<6/450	Yes	No	1C
8	27-G	0.01 (2)	6/450	0.1 (1)	6/60	0.1 (1)	6/60	Yes	Yes	1A
9	27-G	0.3 (0.52)	6/19	0.4 (0.39)	6/15	0.6 (0.22)		Yes	No	1B
10	27-G	0.2 (0.69)	6/30	0.2 (0.69)	6/30	0.2 (0.69)	6/30	Yes	No	1B
11	25-G	0.1 (1)	6/60	0.4 (0.39)	6/15	0.4 (0.39)	6/15	Yes	Yes	1A
12	27-G	0.2 (0.69)	6/30	0.3 (0.52)	6/19	0.3 (0.52)	6/19	Yes	No	1B

BCVA = best corrected visual acuity; FTMH = full-thickness macular holes

**Table 4:** Measures of dispersion of best corrected visual acuity

	Mean	Standard deviation	Range (minimum-maximum)
**BCVA pre-surgical**	0.1825	0.1272	0.01-0.4
**BCVA 6 weeks**	0.2384	0.1255	0.001-0.4
**BCVA 9 months**	0.2967	0.1974	0.001-0.6
**BCVA pre-surgical (logMAR)**	0.9381	0.5517	0.398-2
**BCVA 6 weeks (logMAR)**	0.8192	0.7106	0.398-3
**BCVA 9 months (logMAR)**	0.7699	0.7537	0.222-3

BCVA = best corrected visual acuity

## Discussion

Surgical management of FTMH with poor prognosis presents a significant challenge due to the high rates of closure failure and lower postoperative BCVA compared to idiopathic FTMH [**1,2,4**]. Several surgical approaches have been proposed to improve outcomes, including ILM flap techniques and the use of autologous blood products such as PRGF. Our study aligns with previous findings that blood-derived products can promote FTMH closure by enhancing tissue regeneration and modulating inflammation.

Müller cells play a key role in FTMH formation and repair; however, the exact mechanism remains unclear. When FTMH occurs, the Müller cell cone and ellipsoid zones are disrupted, triggering a complex cell-mediated gliosis response that results in glial scarring, helping restore photoreceptors and the outer layers [**23**].

Several GFs, such as platelet-derived growth factor (PDGF), epidermal growth factor (EGF), transforming growth factor b (TGF-β), and fibroblast growth factor (FGF), activate Müller cells [**24**].

PRGF is a subtype of PRP, enriched with activated autologous platelets, GF, and cell adhesion molecules PDGF, TFG-, EGF, F, and FGF that could play a critical role in macular hole closure by stimulating cellular proliferation and migration, particularly of Müller cells, which are crucial for the repair of retinal tissue [**17,25**].

Although the exact molecular processes by which PRGF promotes macular hole healing are unclear, two potential mechanisms have been proposed: 1) the stimulatory effect on Müller cells that promotes a temporary glial response, and 2) the induction of the contraction of Müller cell processes that promote a concentric contraction of the outer plexiform layer [**23,24**]. Both effects would contribute to the mechanical alignment of the MH edges, thereby promoting closure.

The results of this study showed an anatomical closure rate of 92%, similar to rates reported in other studies. Sánchez-Ávila et al. reported an 87.5% closure rate in eight patients with poor prognosis FTMH (idiopathic, traumatic, myopic, and iatrogenic) treated with PPV and adjuvant membrane PRGF [**17**]. Figueroa et al. analyzed 40 myopic patients with FTMH after PPV with adjuvant iPRGF who were divided into two groups: naïve and persistent FTMH. Interestingly, they reported similar closure rates of 90% and 91% respectively [**26**]. In the present study, the three cases classified as myopic FTMH achieved complete closure. This finding supports the notion that iPRGF can promote closure in myopic FTMH without the need for an inverted ILM flap, especially in cases with a size less than 400 μm.

Additionally, some authors have also studied the inverted ILM flap technique in large FTMH and myopic FTMH and found a closure rate of 94.8% [**27**]. This technique is beneficial because it provides a scaffold for cellular proliferation and aids in hole closure. However, it is difficult to perform in highly myopic eyes or not possible in recurrent or persistent FTMH because the ILM has already been peeled in most of these patients. Therefore, one of the advantages of iPRGF is the simplicity, safety, and shorter surgical time required for this complex FTMH. Other surgical procedures, such as inverted or inserted ILM flap, ART, and hAM, are more complicated to perform, need an experienced surgeon, and take longer.

One notable aspect of our study is the use of the inverted ILM flap technique in combination with iPRGF in four patients with idiopathic large FTMH. All these cases achieved complete closure of the large FTMH. To the best of our knowledge (PubMed search), we have not found any studies that used or compared both treatments for large idiopathic FTMH. A synergistic effect may be expected when these techniques are combined, promoting glial cell proliferation and improving closure rates. Future research should further investigate the combined impact of iPRGF and ILM flap techniques to clarify their potential benefits in this pathology.

Restoration of outer layers was evaluated because this parameter has been demonstrated to serve as a reliable predictor of visual outcome following FTMH surgery [**1**]. Our data revealed that 41% of the cases showed complete restoration of the external retinal layers on the 9-month OCT. Other studies of poor-prognosis FTMH have reported comparable rates, ranging from 21% to 58% [**27,28**].

It is important to note that our analysis treated the recovery of the outer layers as a unified entity, encompassing both the ellipsoid zone (EZ) and the external limiting membrane (ELM), rather than treating them as distinct components. This methodological approach may yield a lower recovery rate than reported in other research endeavors. This may decrease the grade of restoration compared with other studies. Moreover, our results may be limited by the small sample size and the shorter follow-up time in our research, as outer layer restoration can often develop over time [**28**].

Previous studies have shown that MHCP types 1A and 1C have better functional outcomes, whereas type 2 correlates with poorer visual acuity [**21**]. In our study, the most common type of closure was type 1A (41%), and no patient had type 2 MHCP. Typically, type 2 MHCP closure is more frequent when hAM and autologous retinal transplants are used [**21**]. This difference in MHCP could be explained by the iPRGF clot’s transient glial-healing effect on the macula, which favors the regular closure of the fovea. In contrast, methods such as hAM or ART remain in the macular hole cavity for a more extended period, creating persistent glial scars that prevent the normal regeneration of the outer layers of the fovea. In our study, 75% of patients had improved VA, which correlated with most closures by types 1A and 1 B.

Despite these positive results, our study had several limitations. The small sample size, inherent to the rarity of these conditions, and stringent inclusion criteria limit the generalizability of our findings. Additionally, as this was a single-center study, institutional bias may have affected the outcomes. The lack of a control group is another significant limitation, as it prevents direct comparison with other treatment modalities. Future studies should address these limitations by incorporating larger multicenter cohorts and control groups to validate our findings and refine the use of PRGF in clinical practice. Finally, one of the main difficulties with PRP and PRGF is the lack of standardized terminology and the insufficient homogeneity of the elaboration process. An attempt has been made to characterize and classify the numerous techniques currently available for its preparation [**29**].

## Conclusion

In conclusion, this initial study showed positive results in the application of iPRGF for managing poor-prognosis FTMH, thereby establishing a basis for further research to verify its advantages. The efficacy of PPV combined with adjuvant iPRGF has been established to be comparable to alternative methodologies for the closure of FTMH with poor prognosis. This is a relatively simple and time-efficient approach compared to other surgical procedures, which has demonstrated good anatomical and functional results in our cohort of patients with poor prognosis. In addition, the use of iPRGF does not have to exclude other surgical procedures; in some instances, a combination of both can be considered and may be beneficial to the patient. Although the exact mechanism of FTMH closure is not fully understood, the results of the present study support the notion that iPRGF promotes a cell-mediated response that facilitates closure of FTMH with poor prognosis.
